# Abnormal expression of long non-coding RNA rhabdomyosarcoma 2-associated transcript (RMST) participates in the pathological mechanism of atherosclerosis by regulating miR-224-3p

**DOI:** 10.1080/21655979.2021.2023995

**Published:** 2022-01-22

**Authors:** Tao Zhang, Cuina Feng, Xiang Zhang, Bin Sun, Ying Bian

**Affiliations:** a Department of Endocrinology, People’s Hospital of Rizhao, Shandong, China; b Department of Cardiology, Affiliated Hospital of Hebei University, Hebei, China; c Department of Cardiology, People’s Hospital of Rizhao, Shandong, China; d Department of Emergency, Yidu Central Hospital of Weifang, Shandong, China; e Department of General Breast Surgery, Affiliated Hospital of Hebei University, Hebei, China

**Keywords:** Atherosclerosis, ox-LDL, RMST, miR-224-3p

## Abstract

Study shows that long non-coding RNA (lncRNA) plays a regulatory role in cardiovascular diseases, and the mechanism of rhabdomyosarcoma 2-associated transcript (RMST) in atherosclerosis (AS) is still unclear. This study aimed to evaluate the expression of RMST and its possible role in the occurrence of AS. RMST and miR-224-3p level in serum and human umbilical vein endothelial cells (HUVECs) were determined by real-time quantitative PCR (RT-qPCR). In vitro atherosclerotic cell model was achieved by treating HUVECs with ox-LDL. Receiver operating characteristic (ROC) curve assessed the diagnostic value of RMST in AS, and Pearson correlation coefficient estimated the correlation of RMST with carotid intima-media thickness (CIMT) and carotid-femoral pulse wave velocity (cfPWV). Cell counting kit-8 (CCK-8) assay and Enzyme-linked immunosorbent assay (ELISA) were performed to evaluate the effect of RMST on cell viability and inflammatory response. The luciferase analysis was used to validate the relationship between RMST and miR-224-3p. The results showed that in serum and HUVECs, RMST levels were increased, while miR-224-3p level was decreased. ROC curve suggested that RMST had clinical diagnostic value for AS. Besides, CIMT and cfPWV were positively correlated with RMST levels, respectively. In HUVECs, RMST-knockdown notably improved the cell viability and inhibited the production of inflammatory factors. Moreover, miR-224-3p was the target of RMST. In conclusion, RMST has the potential to be a diagnostic marker for AS. RMST-knockdown contributes to the enhancement of cell viability and the inhibition of inflammatory response, which may provide new insights into the conquest of AS.

## Introduction

Cardiovascular disease is one of the major threats to human health and quality of life in today’s society, and atherosclerosis has always been regarded as the number one killer of cardiovascular disease [1]. AS means that under the influence of various risk factors of cardiovascular diseases, lipids in blood are deposited on the damaged arterial wall, forming atherosclerotic plaque, which leads to hardening and narrowing of blood vessels [2,3]. When atherosclerotic plaque breaks, it will lead to blood vessels stenosis and blockage, which is an important pathological basis of cardiovascular diseases [4]. AS is a vascular inflammatory disease jointly regulated by multi-factors [5]. Although significant progress has been made in its treatment, the mechanism of AS still needs to be further elucidated.

In the past few decades, the genetics to explain the pathogenesis of AS has reached a new level. Long non-coding RNA (lncRNA) has been proved to be related to the regulation of the pathogenesis and process of AS [6]. Increasing studies have confirmed that LncRNA participates in regulating the pathological progress of cardiovascular diseases, such as endothelial cell functional, vascular inflammation, and lipid metabolism [7]. For example, the study of Bian et al. showed that overexpression of NORAD reduced the senescence and apoptosis of endothelial cells through the NF-κB signaling pathway and IL-8 and exhibited anti-atherosclerotic effects [8]. Cheng et al. found that LOC285194 is a regulator of proliferation and apoptosis in the development of atherosclerosis [9]. Through RNA-Seq analysis of vascular lesions, Simion et al. found that VINAS was abundant in the intima of aorta and had a role in regulating vascular inflammation [10]. LncRNA rhabdomyosarcoma 2-associated transcript (RMST) located in human chromosome 12q23.1 and showed antitumor activity in some human cancers, such as breast cancer, lung cancer [11,12]. It was reported that the expression of RMST was increased in a cell model that simulates endothelial damage of ischemic stroke, and RMST-knockdown could significantly improve the damage state of endothelial cells [13]. In Zhang et al.’s study, they reported that knockdown of RMST protected mitochondria from I/R damage in animal models of myocardial infarction [14]. However, the role and mechanism of RMST in AS remain to be explored.

Similarly, as a type of non-coding RNAs, microRNA (miRNA) has also been reported to be closely associated with the occurrence of cardiovascular diseases. Lou et al. identified 25 abnormally expressed MiRNAs in a microarray study of atherosclerotic mice [15]. It is reported that the aging-related gene miR-217 plays a role in promoting blood pressure and endothelial dysfunction in AS mice [16]. miR-224-3p, located in the Xq28 region of human chromosomes, has previously been reported to participate in the process of cell malignant transformation and is closely related to the occurrence of a variety of human malignant tumors [17,18]. Recent studies suggested that miR-224-3p also plays a role in human cardiovascular disease. For example, Dharma et al. found that miR-224-3p was associated with coronary microvascular obstruction after angiogenesis in STEMI patients [19]. Wang et al. reported that HDAC1 enhanced the expression of miR-224-3p through deacetylation, and then reduced the apoptosis of endothelial cells and produced anti-atherosclerosis effect [20]. The above research showed that RMST or miR-244-3p is actively involved in the occurrence and regulation of cardiovascular diseases. However, the role of RMST and miR-224-3p in AS still needs more exploratory research.

Based on the conclusions of the above literature, we hypothesized that RMST/miR-224-3p plays a role in the progression of AS. To support our assumption, this study was carried out to investigate the mechanism of RMST in AS. In the current study, we evaluated the clinical value of RMST, and further analyzed the expression and influence of RMST in ox-LDL treated HUVECs through in vitro experiments. Hence, our findings enhanced our understanding of the role of RMST/miR-224-3p in regulation of AS.

## Materials and methods

### Study population and sample collection

A total of 175 subjects were recruited in this study, including 87 cases in the control group and 88 cases in the atherosclerosis group. With the help of 5% of false positive error rate (α = 0.05, two-sided), power = 90%, β = 0.2, 20% of drop-out rate, PASS 11.0 software (NCSS, Kaysville, UT, USA) was used to calculate the sample size. The results showed that at least 79 participants were required for each group to meet the minimum sample size requirement. The inclusion of AS patients followed the 2016 ESC/EAS Guidelines for the Management of Atherosclerosis: 0.9 mm ≤ CIMT < 1.3 mm was considered as the intima of the carotid artery is thickened, CIMT > 1.3 mm was deemed to plaque formation in the blood vessels, and CIMT value <0.9 mm was presented as normal condition [21]. The inclusion principle of the control group followed no history of cerebrovascular disease and the CIMT value was less than 0.9 mm. All volunteers did not include people with a history of serious cardiovascular diseases, including hemangioma, stroke, heart failure, and myocardial infarction. The peripheral venous blood of all subjects was collected, and the serum was obtained after centrifugation, and then stored in −80°C refrigerator for subsequent detection of biochemical indexes. The baseline data and biochemical indexes of the subjects were collected at admission and displayed in Table 1. To verify the diagnostic reliability of RMST in AS, the receiver operating characteristic curve of participants was constructed by GraphPad Prism software, and the values of cutoff, sensitivity, specificity, and area under the ROC curve (AUC) of RMST were also analyzed.

**Table 1.** Basic clinical information of the subjects

Indicators	Control (n = 87)	Atherosclerosis (n = 88)	*P* value
Gender (males/females)	50/37	46/42	0.490
Age (years)	54.76 ± 5.20	55.49 ± 6.31	0.405
BMI (kg m^−2^)	24.86 ± 1.97	25.11 ± 3.28	0.542
TC (mg dl^−1^)	193.52 ± 24.15	198.47 ± 26.64	0.200
HDL (mg dl^−1^)	50.38 ± 4.91	49.17 ± 8.36	0.246
LDL (mg dl^−1^)	120.50 ± 17.73	124.44 ± 18.74	0.155
TG(mg dl^−1^)	147.50 ± 17.60	153.78 ± 25.22	0.058
FBG(mmol l^−1^)	5.30 ± 0.98	5.37 ± 1.10	0.685
SBP(mm Hg)	128.83 ± 17.92	135.41 ± 12.28	0.005
DBP(mm Hg)	82.54 ± 9.78	85.56 ± 9.40	0.039
cfPWV (m s^−1^)	6.46 ± 1.42	11.35 ± 2.94	<0.001
CIMT (mm)	0.63 ± 0.15	1.22 ± 0.16	<0.001

Abbreviations: BMI, body mass index; TC, total cholesterol; HDL, high-density lipoprotein; LDL, low density lipoprotein; TG, triglyceride; FBG, fasting blood-glucose; SBP, systolic blood pressure; DBP, diastolic blood pressure; cfPWV, carotid-femoral pulse wave velocity; CIMT, carotid intima-media thickness. Data are expressed as n or mean ± standard deviation.

The research plan was approved by the Ethics Committee of Yidu Central Hospital of Weifang, and all the subjects who participated in the research had signed the informed consent form.

### Cell culture and cell transfection

Human umbilical vein endothelial cells (HUVECs) gained from American Type Culture Collection (ATCC, USA) need to be cultured with F-12 K medium, 10% FBS, 1% streptomycin/penicillin and survive in an incubator containing 5% CO_2_ at 37°C. In this study, HUVECs treated with Oxidized low-density lipoprotein (ox-LDL) were used to simulate the initial state of endothelial cells during atherosclerosis. According to the previously published method, the gene expression of ox-LDL with different concentrations after treatment for 24 hours was studied [22].

Small interfering RNA against RMST (si-RMST), miR-224-3p mimic, miR-224-3p inhibitor and corresponding negative controls, including si-NC, mimic-NC, inhibitor-NC, were afforded by GenePharma (Shanghai, China). HUVECs were inoculated into a 6-well plate and the cell transfection was achieved by co-incubation with the vector and Lipofectamine 3000 reagent (Invitrogen, USA) based on the product description. After transfection, cells were collected for subsequent studies.

### Reverse transcription quantitative polymerase chain reaction (RT-qPCR)

The expression levels of RMST and miR-224-3p were detected by RT-qPCR, and RT-qPCR method was described in previous reports [23]. Total RNAs extracted from serum or cells were reversely transcribed into cDNA by SuperScript II Reverse Transcriptase kit and PrimeScript™ RT reagent Kit, respectively. Subsequently, using cDNA as the template, the PCR reaction was performed by miScript SYBR® Green PCR kit. GAPDH and U6 were used as internal reference genes of RMST and miR-224-3p, and the relative gene expression levels were normalized to internal reference genes according to the 2^−ΔΔCt^ method. Primers used in this study were as follows:

RMST (forward primer): 5’-AGCAATGCATTCTTTCACAT-3’;

RMST (reverse primer): 5’-CCTGGTGGGTGATGTGAAG-3’.

miR-224-3p (forward primer): 5’-GCGAGGTCAAGTCACTAGTGGT-3’;

miR-224-3p (reverse primer): 5’-AAGCTTGCATCACCAGAGAACG-3’.

GAPDH (forward primer): 5’-CCTCGTCTC ATAGACAAGATGGT-3’;

GAPDH (reverse primer): 5’-GGGTAGAGTCATACTGGAACATG-3’.

U6 (forward primer): 5’-CTCGCT TCGGCAGCACA-3’;

U6 (reverse primer): 5’-GCTTCACGAATTTGCGT-3’.

### Cell viability assay

Cell viability was assessed by cell counting kit 8 assay according to previously published methods [24]. Cells were inoculated in a 6-well plate for cell transfection and ox-LDL treatment. At 0 h, 24 h, 48 h and 72 h after the corresponding treatment, 10 μL of CCK-8 solution was added to the cell culture plate and incubated in dark for another 2 h. Finally, the OD value of cells at 450 nm was measured by a microplate reader (Thermo Scientific, Waltham, MA, USA).

### Enzyme-linked immunosorbent assay (ELISA)

Concentrations of inflammatory cytokines such as tumor necrosis factor-alpha (TNF-α), Interleukin-1β (IL-1β) and Interleukin-6 (IL-6) were determined by ELISA kit (R&D, Minneapolis, MN, USA) according to the previous method with some modifications [25]. Briefly, the samples were incubated with primary antibodies overnight at 4°C. And then, the antibodies were removed, the substrate solution was added and incubated in dark for 45 min. Finally, the stop buffer was added, and the detection was completed within 30 minutes.

### Luciferase reporter gene assay

The online program StarBase 3.0 was used to predict the downstream target gene of RMST, and it was found that RMST and miR-224-3p had complementary binding sites. The 3’-UTR analysis was performed to validate the reaction between RMST and miR-224-3p in accordance with Chen’s study [26]. Briefly, the 3’-UTR fragments of RMST containing miR-224-3p binding site was cloned into pmirGLO vector to construct wild-type (WT-RMST) and mutant (MUT-RMST) luciferase reporter vectors. Whereafter, the HUVECs were co-transfected with vector and miR-244-3p mimic or miR-244-3p inhibitor for 48 h. Ultimately, Dual-Luciferase reporter system (Promega, Madison, WI, USA) was conducted to measure the relative luciferase activity, and Renilla luciferase was regarded as internal reference.

### Statistical analysis

All statistical analyses were performed by SPSS 20.0 software. Kolmogorov–Smirnov test was conducted to evaluate the normal distribution of the measured data, and the data conforming to the normal distribution was represented as mean ± SD. The Student *t*-test was used for comparison between the two groups, and one-way ANOVA was used for comparison among multiple groups. The diagnostic value of lncRNA for AS was analyzed by constructing receiver operating characteristic (ROC) curve. *P* < 0.05 considered to be statistically significant. Each experiment was repeated at least three times.

## Results

### Comparison of baseline data between AS group and control group

The current study recruited 175 individuals, including 87 controls and 88 AS patients. The comparisons of baseline data and clinical indicators between the two groups were shown in Table 1. As we can see from the results that the measured value of systolic blood pressure (SBP), diastolic blood pressure (DBP), carotid intima-media thickness (CIMT) and carotid-femoral pulse wave velocity (cfPWV) in AS patients are significantly higher than those in the control group (*P* < 0.001). Moreover, other indicators such as gender, age, body mass index (BMI), total cholesterol (TC), high-density lipoprotein (HDL), low-density lipoprotein (LDL), triglyceride, and fasting blood-glucose (FBG) did not differ significantly between the two groups (*P* > 0.05).

### The abnormal upregulation of RMST and its diagnostic value for AS

**Figure 1.**
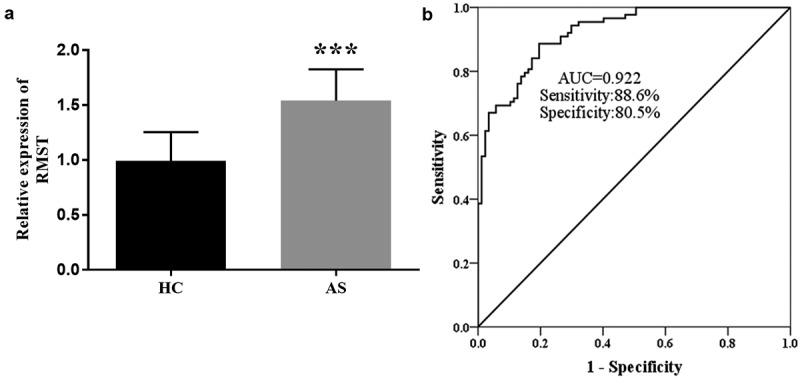
RT-qPCR analysis showed that the expression level of RMST was augmented in AS group. (a) ROC curve exhibited that the AUC value of RMST was 0.922, the sensitivity and specificity were 88.6% and 80.5%, respectively. (b) ***P < 0.001.

To explore the RMST level in AS patients, the expression of RMST in serum samples was detected by RT-qPCR. Figure 1a showed that RMST level was enhanced in AS group compared with controls (*P* < 0.001), suggesting that the abnormal expression of RMST may be related to AS. In view of these findings, we evaluated the clinical diagnostic value of RMST for AS by ROC curve. The results showed that the AUC value of RMST was 0.922, and the sensitivity and specificity were 88.6% and 80.5%, respectively, indicating that RMST has the potential as a diagnostic marker for AS (Figure 1b).

### Correlation analysis of RMST with CIMT and cfPWV

**Figure 2.**
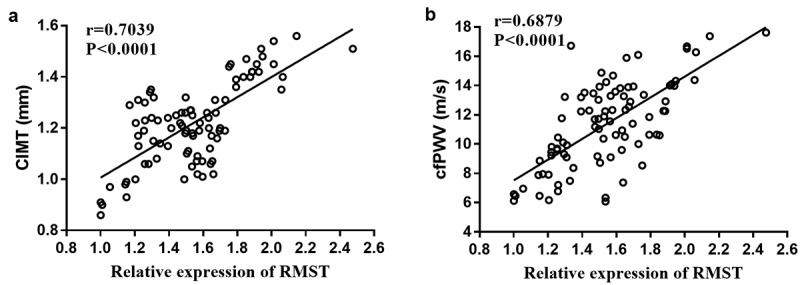
Pearson correlation coefficient analysis of RMST levels with CIMT value (a) and cfPWV value (b).

To evaluate the correlation between RMST with CIMT and cfPWV, Pearson correlation coefficient analysis was performed on the data. It has been confirmed that the elevation of CIMT and cfPWV is closely related to AS. The results are shown in Figure 2a**-b**. We could see that CIMT (r = 0.7039, *P*< 0.0001) and cfPWV (r = 0.6879, *P* < 0.0001) values were positively correlated with RMST expression levels, respectively.

### Effects of RMST expression on cell viability and inflammatory response

**Figure 3.**
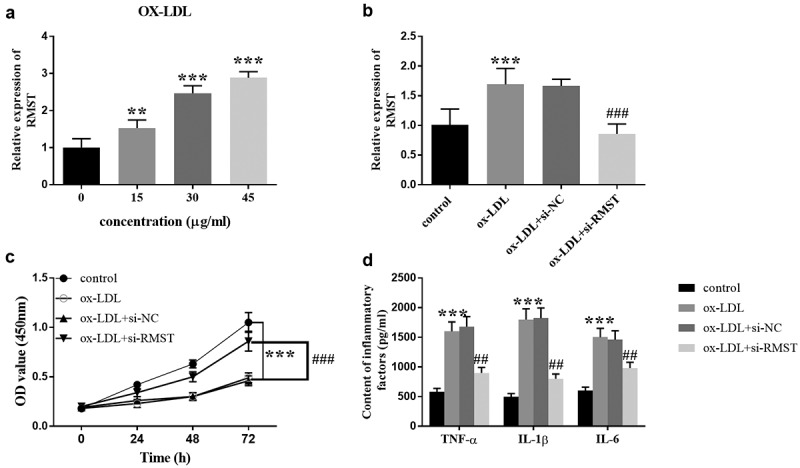
Effects of RMST on HUVECs. The level of RMST in HUVECs treated with ox-LDL at different concentrations. (a) The expression of RMST in HUVECs after transfection of si-RMST. (b) Downregulation of RMST could reverse the inhibitory effect of ox-LDL on cell viability. (c) Suppression of RMST reversed the promoting effect of ox-LDL on production of inflammatory factors. (d) ***P < 0.001, ^###^*P* < 0.001, ^##^*P* <0.01.

To evaluate the possible mechanism of RMST in the development of AS, we constructed an in vitro cell model of AS to evaluate the efficacy of RMST on cell viability and inflammation. Treatment of HUVECs with ox-LDL simulates the state of blood vessels when AS occurs. Figure 3a showed that when incubation time was constant for 24 hours, the expression of RMST in HUVECs gradually increased with the increase of ox-LDL concentration. When ox-LDL concentration was equal to or greater than 30 μg/mL, RMST expression level was significantly higher than that of control group (*P* < 0.001). Therefore, 30 μg/mL ox-LDL was selected as the treatment concentration of ox-LDL in subsequent experiments. Figure 3b revealed that transfection of si-RMST inhibited the enhancement of RMST induced by OX-LDL treatment in HUVECs (*P* < 0.001). For cell viability, the HUVECs viability was significantly inhibited with the addition of ox-LDL. However, OD value of cell viability was distinctly upregulated after RMST knockdown (Figure 3c, *p* < 0.001). Furthermore, ELISA results also showed that the cells transfected with si-RMST could clearly reverse the promotion efficacy of ox-LDL on the production of inflammatory factors (Figure 3d, *p* < 0.001).

  

### Analysis on downstream target genes of RMST

**Figure 4.**
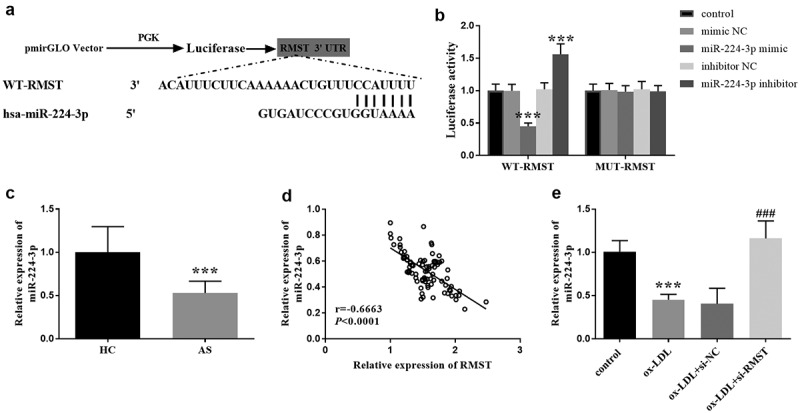
Complementary sequences of RMST and miR-224-3p. (a) The targeted relationship between RMST and miR-224-3p was evaluated by luciferase reporter gene assay. (b) RT-qPCR analysis showed that the expression level of miR-224-3p was attenuated in AS group. (c) The linear relation between RMST and miR-224-3p in serum was carried out by Pearson correlation coefficient. (d) RT-qPCR analysis revealed that the level of miR-224-3p was decreased in ox-LDL treated HUVECs. (e) ***P < 0.001, ^###^*P* < 0.001.

In order to explore the mechanism of RMST in vitro, we predicted the target of RMST by bioinformatics method, and verified the interaction between RMST and its target by luciferase reporter gene. The binding sites of RMST and miR-224-3p was presented in Figure 4a. And in Luciferase reporter gene assay, the luciferase activity in WT-RMST group was decreased after HUVECs were transfected with miR-224-3p mimic, which was not observed in MUT-RMST group (Figure 4b, *p* < 0.001). In the serum samples, we could see that the level of miR-224-3p in AS patients exhibited a marked downward trend in comparison to controls (Figure 4c, *p* < 0.001), and additionally, we found a negative correlation between RMST and miR-224-3p in serum samples in AS patients (Figure 4d, r = −0.6663, *P* < 0.0001). Moreover, miR-224-3p level was decreased in ox-LDL treated HUVECs. Similarly, RMST knockdown dramatically enhanced the level of miR-224-3p (Figure 4e, *p* < 0.001).

### Effects of miR-224-3p expression on cell viability and inflammatory response

**Figure 5.**
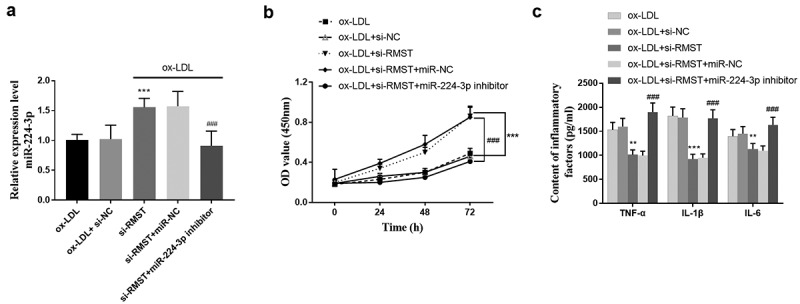
Effects of miR-224-3p on HUVECs. The expression level of miR-224-3p in HUVECs after transfection of si-RMST and miR-224-3p inhibitor. (a) Downregulation of RMST and miR-224-3p could decrease the enhanced cell viability induced by RMST knockout. (b) Simultaneous inhibition of the expression of RMST and miR-224-3p counteracted the inflammatory inhibition induced by RMST knockout. (c) ***P < 0.001, **P < 0.01, ^###^*P* < 0.001.

Considering the interaction between miR-224-3p and RMST, we evaluated the efficacy of miR-224-3p on the cell viability and inflammatory factors of HUVECs after inhibiting the expression of RMST by in vitro cell transfection method. The results showed that miR-224-3p expression was up-regulated after transfection of si-RMST into cell models. Meanwhile, the increased miR-224-3p level due to RMST inhibition was offset due to transfection of miR-224-3p inhibitor (*P* < 0.001, Figure 5a). Cell viability assay revealed that the enhanced cell viability due to RMST knockdown could be canceled in the presence of miR-224-3p inhibitors (*P* < 0.001, Figure 5b). In addition, the test results of inflammatory factors showed that the reduction of RMST had an inhibitory effect on inflammatory factors, while the original inhibitory effect on inflammatory factors disappeared after both RMST and miR-224-3p were resisted (*P* < 0.001, Figure 5c).

## Discussion

AS is a chronic inflammatory reaction in the arterial wall caused by lipoproteins, immune cells, pro-inflammatory factors, and extracellular matrix components that are deposited under the arterial wall after degradation and necrosis [27,28]. AS is the main pathological inducement of cardiovascular disease, and the most common cause of coronary artery disease, carotid artery disease and peripheral artery disease [29]. The occurrence of AS is characterized by the formation of AS plaque [30]. It is reported that among the numerous cardiovascular risk factors, the increase of plasma cholesterol level may be unique, which is enough to promote the development of atherosclerosis even in the absence of other known risk factors [31]. Therefore, in many studies, HUVECs induced by ox-LDL in vitro are usually used to construct AS cell model. In the present study, we could see that the expression of RMST is enhanced in the serum of AS patients and ox-LDL treated HUVECs, and the highly expressed RMST demonstrated excellent clinical diagnostic value for AS. In vitro, it was observed that RMST knockdown can not only ameliorate the inhibition of cell proliferation induced by ox-LDL, but also reduce the inflammatory response, which may be achieved through targeted regulation of miR-224-3p expression.

RMST is a 2100nt lncRNA found in the gene region of rhabdomyosarcoma, which plays an important role in the pluripotency and neural differentiation of embryonic stem cells. Previous studies on RMST focused on tumors, while few studies reported the role of RMST in other diseases. For example, Simona et al. reported that RMST was found to be downregulated in triple negative breast cancer (TNBC), confirming that RMST exerted anti-tumor efficacy by inhibiting the proliferation, migration, and invasion of tumor cells [32]. In recent years, RMST has been found to have significant effects on the atherosclerosis-triggered diseases. Hou et al. found that RMST was increased in the blood of patients with ischemic brain injury, and intraventricular injection of RMST inhibitor could reduce the area of cerebral infarction and improve the neurological function [33]. Zhang et al. showed that RMST was upregulated in a mouse model of myocardial infarction [14]. Although the subjects of the above study were not AS cases, AS is a cause of the occurrence of these diseases. Previous studies have provided important insights and underpinned our current research. Our study revealed that the expression of RMST exhibited an enhanced trend in the blood of AS patients. The clinical significance of CIMT and cfPWV detection in cardiovascular diseases is obvious to all. It was noteworthy that CIMT and cfPWV of AS patients are significantly positively correlated with RMST levels in the present study, which further verified the relationship of RMST and AS. In addition, this study successfully simulated the status of endothelial cells during AS through ox-LDL treatment in vitro, and through this model, we confirmed that inhibition of RMST could improve ox-LDL-induced inhibition of cell viability and enhancement of inflammation. Theoretically, these results may provide a new direction for explaining and elucidating the mechanism of AS.

In recent decades, the research on lncRNA act as a competitive endogenous RNA (ceRNA) can be used as a miRNA sponge to combine with miRNA and play a role in gene regulation has increased year by year [34]. Therefore, we speculated that RSMT may co-regulate AS with some miRNAs. As expected, we predicted that miR-224-3p was the downstream target gene of RMST through bioinformatics analysis techniques. And our study showed that the expression level of miR-224-3p was significantly reduced in AS patients and in vitro AS cell models, and the level of miR-224-3p was negatively correlated with RMST. These results preliminarily showed that RMST may regulate cell viability and inflammatory response through targeted regulation of miR-224-3p in AS cell model, and thus regulate the process of AS. The association between miR-224 gene and cardiovascular disease has always been a research hotspot. In a study by Hu et al., down-regulation of miR-224 promoted the formation of vulnerable atherosclerotic plaques and accelerated vascular remodeling in coronary syndrome by activating the TGF-β/Smad pathway [35]. Of note, although the relationship between miR-244 and AS is not completely clear, its relationship with NF-kB, WNT, TGF-b and other signaling pathways has been gradually confirmed, and they are all related to atherosclerotic signaling pathways [36]. Beyond that, the interaction between miRNA and target genes should also be paid attention to in exploring the mechanism of action of miRNA. miRNA regulates the expression of target genes by cutting or inhibiting the translation of target gene transcripts, thus completing the gene regulation of various life activities. In Wang et al.’s study, they found that miR-224-3p inhibited the expression of FOSL2 through deacetylation of HIF1α in vitro model, which inhibited endothelial dysfunction and cellular inflammatory reaction, thus showing a good anti-atherosclerosis effect [20]. Like other studies, this study also has limitations that cannot be ignored. Among them, the most obvious deficiency is the lack of systematic in vivo experiments in this study. We should conduct the in vivo experiments to verify our cytological findings by establishing animal models of atherosclerosis. Another limitation is that this study was conducted in a single-center clinical setting with a small sample size, which may lead to selection bias. In the future, we still need to conduct multi-center studies with large random sample size to further verify our results.

## Conclusion

To sum up, this study has systematically studied and expounded the role and regulatory mechanism of RMST in AS by clinical sample analysis and in vitro cell assay. In ox-LDL-treated HUVECs, the inhibition of RMST promoted the cell proliferation and inhibited inflammation via targeting miR-224-3p. The regulation mechanism of RMST and miR-224-3p in AS was expounded, which opened a new perspective of taking RMST as a therapeutic target to conquer AS. Therefore, we preliminarily speculated that RMST could be a new therapeutic target in AS.

## Highlight

- In AS patients, the expression of RMST was increased, while miR-224-3p was decreased.
- RMST showed high diagnostic accuracy for asymptomatic AS.
- Knockdown of RMST increased cell viability and inhibits inflammatory response in HUVECs.
- RMST may play a regulatory role by targeting miR-224-3p.
